# Rethinking sports history to include sportswomen in 1900s France

**DOI:** 10.3389/fspor.2023.1185638

**Published:** 2023-07-31

**Authors:** Florys Castan-Vicente

**Affiliations:** ^1^ L-VIS, STAPS, University Claude Bernard Lyon 1, Villeurbanne, France; ^2^ CHS, History, University Paris 1 Panthéon-Sorbonne, Paris, France

**Keywords:** sports history, women’s history, gender studies, professionalism, feminist studies, France

## Abstract

In the history of French sport, the practice of physical activities by women is essentially considered non-existent before the 1920s, with the exception of a few aristocratic women. Although this idea persists, it has been challenged by recent research on early sportswomen. These studies raise the question of the scope of sports history, and indeed the very definition of sports itself. These are usually defined in the social sciences as physical activities that are organized, codified and institutionalized, structured by clubs and federations. While at the beginning of the 20th century these clubs and federations were most often closed to women, this does not mean that women were not practicing sports. Physical activities were gaining increasing popularity among women even before this time, and not only among the upper class. There is evidence of women swimming, cycling, racewalking, and even wrestling or boxing in the United States, Canada and Britain, as well as in France. These practices necessarily developed outside institutions, with women taking them up as individual pastimes. As demand grew, some sought to profit from this, and sports promoters organized the first competitions. Journalists then reported on these events in the press, sometimes with amusement, sometimes with disapproval.

Yet the first women walkers, runners, cyclists and other athletes are only now beginning to appear in historical studies. This paper seeks to contribute to the rehabilitation of these sportswomen, who include working-class boxers and wrestlers, all of whom have long been subject to a double exclusion—institutional and historical. It presents the history of the first competitions of sportswomen—professional or amateur—in France at the turn of the century, a first foundation stone in writing a new and more inclusive history of sport.

## Introduction

In the history of French sport, the practice of physical activities by women is essentially considered to be non-existent before the 1920s, with the exception of a few aristocratic women. For example, Thierry Terret, specialist on gender in sport, notes in his reference work on the history of sport (republished in 2023): “Sports practitioners are men, and with a few exceptions, for example in swimming, tennis and mountaineering, women are absent from sports organizations. Les Femmes de Sport, the valuable work published by Baron de Vaux in 1885, gives only a few examples of aristocratic women whose social isolation limited theirach to serve as models. The few pioneers who embarked on cycling created a scandal and were the object of derision in the press” (1).

While this is undeniably true, it only partially describes the extent of French women's physical barely taking into account experiences outside sports. In 2006, Catherine Louveau, another specialist in gender issues wrote: “Like sports practices and institutions, the histoy of sport in recent decades has not considered gender. Without stating this explicitly, this history concerns in reality men and the masculine.”

Yet she herself downplays women's participation in sport, claiming that sport was “a hobby of aristocratic and socialite women” (2) until the first women's sportsclubs were established in the 1910s.

Although this idea still persists today, it has been challenged by recent research on early sportswomen. These studies raise the question of the scope of sports history, and indeed the very definition of sports itself. These are usually defined in the social sciences as physical activities that are organized, codified and institutionalized, structured by clubs and federations. Yet at the beginning of the 20th century, these clubs and federations were most often closed to women (and sometimes to the working class) (3), with this androcentric institutionalization excluding women from the history of sport. In fact, physical activities were gaining increasing popularity among women even before the 1900s, and not only among the upper class. There is evidence of women swimming, cycling, racewalking, and even wrestling or boxing in the United States, Canada and Britain, as well as in France. These practices necessarily developed outside institutions, with women taking them up as individual pastimes. As demand grew, some sought to profit from this, and sports promoters organized the first competitions. Journalists then reported on these events in the press, sometimes with amusement, sometimes with disapproval.

Yet the first women walkers, runners, cyclists and other athletes are only now beginning to appear in historical studies. M. Ann Hall, a Canadian pioneer of feminist sport studies, recently published a work on the first female American professional cyclists (in the 19th century), once dismissed as simply “salacious entertainment” (4). In France, Philippe Tétart has brought to light one of the country's first women cycling champions in the 1890s (5).

This paper seeks to contribute to the rehabilitation of these sportswomen, who include working-class boxers and wrestlers, all of whom have long been subject to a double exclusion—institutional and historical. It takes an intersectional approach, as some of these women were also viewed through a prism of race or class as well as gender. It presents the history of the first competitions of sportswomen—professional or amateur—in France at the turn of the century (late 19th century to the 1930s), a first foundation stone in writing a new and more inclusive history of sport.

The first women's sports events are scarcely visible outside of the generalist or sport press. The press followed these events, sometimes organizing the competitions themselves, and then used their media platform to report or polemicize on the subject. To investigate the history of women in sport in France, this study examined articles in the press on major competitions, as well as antifeminist essays from the period, which demonstrate both the delegitimization of women practicing sport and their sexualization—a parallel process. Writings of the sportswomen themselves, showing their struggle for recognition, were also examined, although these are unfortunately rare.

## Discussion

### Women in sport in the eyes of the French media in the early 1900s

One of the earliest known women's sports events in France was the *Marche des Midinettes* in 1903. Organized by the newspaper *Le Monde Sportif*, the event was a 12-km walking race from Paris to Nanterre for “*midinettes*,” a term referring to young women working as seamstresses or assistants in the fashion industry in Paris. The race received a lot of press attention, mainly in an ironic tone. For example, the daily newspaper *L’Éclair* wrote that the event attracted many “*marcheuses d’un autre genre*” (6), (“walkers of another type,” with *marcheuse* taking the double meaning of “streetwalker”). It referred to the women's coaches as “*entraîneurs*” (a word that means “trainer” as well as “pimp” in French). Such ironic references drew an implicit connection with sex work, a process of sexualization that delegitimized these working-class women rather than considering them in the nobler sense of sportswomen. This race took place at a time when there was a lot of discussion generally and in the press about the place of women in sport. At the time, French athletics organizations were reserved for men. As a way to make headlines and attract the attention of the general public, Paul Rousseau (7), the director of *Le Monde Sportif* and the founder of the first federation of cycling and of boxing, organized this racewalking competition (8). It fit into the category described by the ethnologist Anne Monjaret as above all “popular playful events,” or even “street shows,” organized by and on behalf of the press (9). This event can be seen as characteristic of the type of competitions open to women for several decades, and sometimes even today.

In an article in *L'Auto* written the day before the race, the *Marche des Midinettes* was used to evoke the larger question of women in sport, arguing that “the modern woman” should remain a good “machine for children” and not neglect “her most fundamental role:” that is, to stay fit in order to have children (7). But a woman that trained too much or became a professional athlete would lose her “charm” (a word that also refers to “allure” or “appeal” in French). The journalist concluded that the idea of the race was “amusing,” attracting both those interested in sport and those whose only demand of women was that they “stay alluring.” So even before the race took place, we see it raising questions containing structuring elements of antifeminist discourse: a woman's role as a mother, her duty to be in good health for childbearing, her role as a wife who must not neglect her domestic tasks, and finally, the imperative of beauty. After the competition, the same publication (*L'Auto*) reported that there were between 300,000 and 500,000 “curious spectators” in the crowd, concluding that it was a “big, very big, huge success for curiosity,” going into detail regarding the outfits and physique of the contestants, with a suggestive reference to the “loud libidinous laughter” of the spectators (10). Another more bourgeois sports paper, *La Vie au Grand Air*, went even further in its description of the spectators' outbursts, depicting them as characteristic of a detestable working class: “A band of vile, walleyed ruffians with flat, greasy hair and bow legs appeared almost immediately, draped in miserable rags. All the gangrene of the street was there, throwing dirty jeers at the troop of light-colored bodices and flimsy blouses” (11).

Using the pretext of complaints about the event made by some of the contestants, *L'Auto* decided to steal the idea from its competitor, *Le Monde Sportif*, and organize a 300-m race as a second event. This would also result in mockery from the press, with *La Vie au Grand Air* publishing photographs with ironic titles: for example, a photograph of runners putting their hair back up was captioned “What will henceforth be called a sports scene” (12). This irony not only devalued these amateur sportswomen, but often involved sexualization. One photograph taken in the middle of a race and revealing a runner's petticoats was captioned “Horrible details!”

These first competitions were a success for the sports press, with consequent financial rewards. This led to newspapers regularly rekindling controversy around women practicing sport, with women athletes caught in the middle of the debate. *L'Auto* in particular spoke out alternately for and against women's competitions depending on where it saw its financial interests. The planned events might include a beauty contest, in the aim of attracting the public through women's physical attributes. In this way, the press presented sportswomen solely through the lens of the spectator, without considering the point of view of the aspiring athletes themselves. The conventional discourse thus focused on the physical appearance of the sportswomen under the gaze of the press, the organizers and the spectators; but what meaning did these events hold for the participants themselves? While there is a glaring lack of sources regarding this, the fact that some of the contestants trained to win shows that other meanings may have been possible for at least some of those participating.

The ironic distancing and sexualization of female practitioners by the press are classic rhetorical devices for belittling and undermining their actions. They discount the possibility of a quest for sporting performance or a demand for true athletics, making these ambitions invisible by treating the incongruity of the event as comical. In the absence of “regular” or “official” competitions organized by sports federations, sportswomen were subject to the aims and interests of sports promoters, who were often also media owners. As shown by Philippe Tétart, this marginalization made sportswomen at the time very vulnerable to unscrupulous managers and other threats (9). As an example, he cites suspicions of doping among female cyclists at the turn of the century. These dangers were all the more visible in “high-risk” sports.

In fin de siècle France, the first competitions open to women were racing, cycling, swimming (13) and winter sports (skiing and bobsleigh), as well as aviation and motorsports. While winter sports were reserved for the upper classesthis was not the case for other activities. In 1897, Pierre Laffitte, director of *La Vie au Grand Air* and the women's magazine *Femina*, organized a motorbike competition; in 1898, a “cycling-skates” (early precursors to roller skates) race; and in 1902, a challenge for women aeronauts (in dirigible balloons). In 1910, the *Femina* aviation cup was won by Hélène Dutrieu, a professional sportswoman from the working class (14): at the age of 14, she had become a professional cyclist in order to earn a living (15). She later took up the motorbike, performing an act in which she raced down a long inclined track before sailing into the air, and then decided to try her hand as an aviator (16). In 1912, after winning an aviation event twice, she protested in the press, asking for a real competition rather than a flight-time challenge circling over an airfield, which she considered more dangerous because it was so boring it was likely to put pilots to sleep (17). She called for women aviators to be considered on an equal footing with men, to be able to compete in a real competition, and to be better protected from risks. The first women's world bobsleigh champion in 1910, skier, cyclist and aviator Marie Marvingt also recounted her regular brushes with death (18).

The press was also involved in organizing cycling races: Néva (the penname of Madame Maujard) was both a professional cyclist and a journalist with the feminist newspaper *La Fronde* (the term means “revolt” in French). In her articles, she defends women cyclists, “who have been under attack for some time, precisely because they are the first to want to free themselves from the old prejudices that prohibit women from a career in sports” (19). She took a stand for the professionalism of women cyclists, supported the formation of a union and reported the increasing level of training and performance of women in the sport (20).

But these voices of sportswomen speaking for themselves are rare—particularly those from the working class, who rarely left written records of their experiences. When testimonies from sportswomen of the time do exist, they bear witness to women committed to their practice: sometimes to the point of mortal danger, as with female aviators. Those from different social classes than the bourgeoisie may have been partly motivated by the income from their practices. Some campaigned to win recognition of their presence, to legitimize their practice, and to argue for their performance to be fairly rewarded. Opposition to these demands is evidenced by discourse emanating from the sports press or from antifeminists of the time.

### Depicting the sportswoman as exhibitionist

Around the turn of the century, a great number of articles decried the scandalous display of scantily clad women. These reactions can be described as antifeminist, in the sense that this is defined as a doctrine that opposes feminism: “feminists and antifeminists each embody a logic that is antagonistic and that interacts dialectically,” each trying to convince public opinion and make society evolve the direction it wants (21). As antifeminism rejects the emancipation of women, sportswomen come under fire as active “modern women” who challenge a cornerstone of the hierarchy between the sexes: women as the “weaker sex.” The antifeminist polemics around this can be virulent. French sources from the late 1800s to early 1900s reveal the paradox noted by Philippe Tétart: while sportswomen are criticized for being too sexual, at the same time they are vilified for being unattractive. Or they are accused of being gender-neutral “women-men” who reject their “natural” role: for example, in a postcard from around 1900 that depicted a female cyclist in trousers going for a ride and leaving her husband to look after a crying newborn (22).

Sportswomen and feminists were jointly accused of rejecting femininity, and with it the functions devolved to women: domestic tasks and, above all, motherhood. These antifeminist arguments were prevalent in the press and in popular culture. Sportswomen regularly found themselves hypersexualized; this was particularly the case for cyclists, who had to wear bloomers that exposed their calves. At the same time, they were accused of becoming masculine, imitating men and abandoning their homes. The antifeminist writer Octave Uzanne expressed this fear of non-differentiation, crystallized in sportswomen and cyclists in particular: “every day the battalions of gynandrous Parisian women increases, these creatures making themselves virile through training, who would willingly say about themselves […]: ‘Look, I’ve turned myself into a man’” (23). Similarly, the journalist John Grand-Carteret did not differentiate between sport and feminism. He indiscriminately uses “new women,” feminists, trouser wearers and sportswomen as synonyms. The “woman in trousers,” whether fencing or cycling, was viewed as a “neuter gender,” a person who is “half man half woman” (24). In his book on the subject, he represents “a woman in the year 2000” in short pants, holding a cigarette and carrying a walking stick, attributes that were seen as male (24). The bicycle is seen as the symbol of this evolution in dress toward a less marked differentiation between the sexes: from an antifeminist perspective, toward uglification. A cyclist in bloomers is considered unattractive, too fat or too thin, “asparagus or tobacco jar” (tall and skinny or short and round) (24).

These sportswomen nonetheless attracted interested eyes: for a male sports journalist of the time, a situation lending itself to tragicomic farce (23). One reported that a provincial old man arriving in Paris died of a fit of modesty after passing a young female cyclist (25). The journalist himself seems to fantasize about the “tight-fitting jerseys” and the bare flesh of women runners, describing “a clavicle protruding through the cotton jersey,” or in a bizarre food reference, “a tibia whose flesh seemed to have been scraped off with a knife, as when eating a chicken drumstick.” He considers the women's cycling competition that has been announced an aberration, urging male cyclists to “*chauffer ces chauffeuses*” (“cause trouble for these troublemakers”), using a play on words that likens women drivers to gas heaters (*chauffeuses*) and alludes to *pétroleuses*, a term used for women agitators (26).

This accusation of debauchery is a process that uses eroticization to undermine women's actions. Studies in France have argued that certain erotic postcards were a means of ridiculing women seen as performing male functions (27). In 1910, the year in which the first aviation license was issued to a woman, a postcard was published showing a *midinette* drawn in a sexualized manner, with an arched back astride a monoplane, her legs uncovered up to her knees and her frilly underwear exposed, being chased by another plane piloted by an old bourgeois man (28). This cartoon thus does double duty, making fun of both the pretensions of the *midinettes*, whose first race took place in 1903, and the first female aviators.

Early sportswomen faced other types of accusations, such as that of “aping men.” Theodore Joran was a widely known antifeminist French journalist and writer of the early 20th century. On the subject of women breaking the mold he wrote: “Why do they gamble on racing, pretend to have an interest in sports they will never understand, try to speak slang, smoke, dress in male attire, and embark on gender-inappropriate careers such as journalism and reporting? Why? To ape men, plain and simple” (29).

In a similar way, the press was prone to describing how women's events “ape” men's, but cannot be as important (8). They were viewed as “exhibitions”—only men's sport was considered a serious, legitimate business. This point of view, which can be placed politically in the antifeminist camp, is sometimes found in the history of sportswomen. This may be partly because sources on these first practitioners are rare, for the most part consisting of articles in media (8). Then as now, the media often sought out controversy to boost sales, and accusations of immorality against sportswomen seemed to be effective at whipping up indignation regarding an event organized by a rival newspaper. These turn-of-the-century media sources therefore contain significant bias: gender bias and commercial bias. Philippe Tétart takes the example of the articles of G. de Moncontour, “editor of *La France Cycliste*, vice-consul of the Union Vélocipédique de France (the main cycling union) and president of the Northern Coast Cycling Club,” who in 1891 described women's cycling competitions as “exhibitions of *cocottes*” a term that can refer to hens, cheap perfume or, more pejoratively, prostitutes (8, 30).

er stereotypes rather than “challenged” them. But one could ask why these events should be interpreted as not sport, rather than as an attempt to play sport in a context of institutional exclusion and resulting in a violent reaction to reinstate norms? In fact, testimonies of various sportswomen of the 1900s recounting aggressions seem to indicate the latter. ms. This type of analysis, still common in sports history, seems partly responsible for rendering invisible the origins of the history of sportswomen, which is often seen as starting in the 1920s or, for some historians, even as late as the 1960s. Yet the reality is that, excluded from federations and official competitions, the first French sportswomen had no other opportunity to practice sport than commercial competitions, often organized by the press. They were not allowed access to physical activities in the same way as men, according to the canonical and “noble” view of sport and competition as they were defined at the time, and which still persists today in sports science. Sportswomen were presented as ridiculous, if not indecent, for practicing an activity seen as far removed from federation sport and not considered legitimate. These competitors struggled to be taken seriously as true sportswomen by the commentators of their time, despite their demands. As the main source on early sportswomen available consists of press articles, historical research has tended to reproduce this discourse uncritically. Women's exclusion from institutional sport, resulting in early sportswomen being perceived as mere entertainment for the public, still has historical consequences today. The first sports events open to women often continue to be considered not as real competitions, but as events with an objective of spectacle rather than a quest for sports performance or breaking records.

### Toward a more inclusive definition of sport?

It can be argued that this institutional exclusion that prevented women's sport from being taken as a serious, legitimate “real sport” is reflected in the historical exclusion of women in sport still observed today. This is even more true for working-class sportswomen, whose point of view is rarely recorded. Some are even excluded from the contemporary definition of “sport” by the social sciences.

For example, boxing and wrestling, as combat disciplines, have historically been considered masculine and very transgressive for women. Despite this, around 1910 boxing became fashionable for French upper-class women, but was practiced in secret, away from prying eyes, for the purpose of physical conditioning and not for combat. Its practitioners, who took lessons from renowned champions, preferred not to mention their names (31). This activity was considered to have come from the United States, influenced by feminism (32).

Beyond those practicing boxing discreetly, there are records of public shows by female boxers and wrestlers in France from the middle of the 19th century onwards. Several troupes existed, in Rouen from 1868, then at the Folies-Bergères, whose female wrestlers were managed by the champion Pietro (33). In sources from the time, they are colorfully described, said to undergo rigorous training and covered in bruises; these working-class women were reported as often married to “strong men or acrobats” or occasionally as widows or “free women.” Some were described as having tattoos, fiery hearts emblazoned with their lover's name (34). On the posters announcing their performances, they are shown scantily clad, wearing sleeveless shirts and short shorts, in front of an all-male audience of supporters (35). The posters in the archives show only white women and do not mention the wrestlers' names. However, certain stage names were recorded, bearing witness to the diverse origins of these female wrestlers, as well as to their racialized or sexualized presentation: the “street singer of Batignolles” against the “lioness of La Chapelle,” the “voluptuous” Sandra Porter against the “sassy” Norah from Martinique, the “wild one” against the “negress” (33). The twins Natta and Karlotta Kiddjah were described as wrestling barefoot in short skirts and tank tops (33). These stage names indicate that wrestling brought together women from the working class in Paris and from overseas. The descriptions also indicate that these wrestling shows were depicted as falling between combat and eroticization; for non-white or racialized women, this eroticization was coupled with exoticization, in a dynamic that has been described as the “spectacularization of the black body” (36). The existence of these troupes at the turn of the century can be linked to the emergence of the entertainment industry, boosted by the popularity of Parisian performance halls, although this was more the case in North America than in France (37). The flourishing entertainment industry also had bridges with other sports besides wrestling. The famous swimmer Annette Kellerman had a career in both sport and entertainment, which was socially accepted, as swimming was considered a sport fitting for women (13). But the working-class women in combat sports were considered entertainers rather than sports figures and likened to music-hall artists, objects of fantasy and mockery excluded from the category of “women's sports:” “We will, of course, leave aside the music hall performers, tightrope walkers, contortionists, animal tamers and weightlifters whose activities do not belong in a book devoted to women's sports. Sport should be considered an enjoyable exercise practiced to attain physical fitness. While those ladies may sometimes interest us, they never amuse us. They veer into excess and their exhibitions are neither sporting nor feminine” (31).

These early female wrestlers and boxers had a bad reputation, viewed as less than respectable, in some cases as similar to sex workers; in an account of women in sport in the 1930s, their bouts were described as “pornographic exhibitions” (38). In contrast, male wrestlers of the time who also performed in theatres had an official arena in which to compete as well, a “championship” that crowned “champions” and garnered headlines in the sports press: regardless of putting on public shows, they were considered fully-fledged sportsmen, respected and respectable (39). Female boxers and wrestlers of the period found little support, even among the most sports-enthusiastic feminists. In the context of women boxers taking part in a music hall show, which the press described as a “victory of feminism,” the contemporaneous French journalist Jane Misme begged to differ (40): while feminism sought equality, including in boxing, she voiced her disapproval of these “barbaric spectacles,” which rather than a “triumph, are a deviation from feminism.”Yet women's exclusion from sports institutions, leaving no option but to train or compete as amateurs, made it impossible for female wrestlers or boxers to practice their sport in a context other than that of entertainment, which was also the only remunerative option. For working-class women, these shows were an opportunity, a means of transforming their physical capital—their strength, skill and appearance—into economic capital. But they troubled feminists and middle-class and upper-class sportswomen who wanted to avoid the stigma of women of ill repute. In her history of feminism from 1914 to 1940, historian Christine Bard points out the absence in the “feminist pantheon” of female singers and actresses, who also put their bodies on display for pay (41). To this list can be added dancers, as well as wrestlers and boxers, all of whom were seen as incompatible with the feminist movement's quest for respectability.

Moreover, they were excluded from feminist demands. Working in an environment with a dubious reputation, likened to sex work in public opinion, these working-class, sometimes non-white, professional wrestlers and boxers were not considered sportswomen, and are invisible in the history of French sport, despite the North American dynamic launched by Roberta Park with her study of turn-of-the-century cyclists and walkers in America (42). Paradoxically, while these professional sportswomen were treated as feminists by the press, feminists did not consider them sportswomen—or even respectable. In addition to the stigma of prostitution, female wrestlers and boxers were put in the same box as circus strongwomen, to whom feminists regularly found themselves compared to their chagrin.

The history of these professional sportswomen in France thus has yet to be written. As the press of the time tended to focus on high society sportswomen (42), sources are scarcer but are not non-existent, and it is important not to “let it lie upon the table,” i.e., not to let the history of less normative sportswomen be overlooked (43). This area merits further study, which may change our view of sporting “firsts,” often limited to the upper classes, and to reevaluate the distinction between sports considered “serious” and “respectable” and entertainment—events that belong more to the world of show business and associated with the masses. Excluded from traditional definitions of sport, women wrestlers and boxers were marginalized during their lives, just as today they are marginalized by sport history. Including them at last in the written history of sport would involve changing the definition of “real sport” inherited from Pierre de Coubertin. As social and cultural historian Patricia Vertinsky stated, historians should “shift the sport historian's gaze from Pierre de Coubertin's aristocratic vision for the making of men through sport and physical culture in fin de siècle Europe, to the Europe that embraced Isadora Duncan's modern dancing and feminist politics and its accommodation to new approaches to movement and physical expression” (44) (2010: 20).These different strands of scholarship that endeavor to unpack the myth of modern sport have much to offer a more inclusive history of sport. Broadening the definition of sport to dance-related activities such as Irène Popard's “rhythmic gymnastics” seems essential to this (45), but it could arguably be widened even further to include women wrestlers and boxers and others excluded from “real sports” and not taken seriously as athletes.

## Conclusion

Historically, the first sports competitions open to women in France were driven by commercial and media demands rather than organized by sports federations; institutionalization did not take place until the First World War. These early contests were primarily races (walking or running, swimming, cycling, rowing, automobile/motorbike/plane racing). Organized by and for the press, and by men, these far from independent events nonetheless often received the support of militant feminists. Feminists also participated in them, as in the case of Néva. The competitors made demands to be taken seriously: the *midinettes*, for example, protested against the poor organization of their race, while female aviators, who had greater cultural and political capital, mobilized against competitions that they considered overly infantilizing, demanding greater recognition of their abilities and fairer treatment compared to their male colleagues. The turn of the century thus saw the first calls against competitions that were male-controlled, instrumentalized (*La Marche des Midinettes*), “softened” (the Femina aviation cup) or excluding (automobile races).

Today the sportswomen that competed in these first women's events remain invisible within the history of sport—even those who were famous for their performances during their lifetime. As Ann Hall argued for professional 19th-century cyclists, it seems essential to rethink categories and topics of study to include these athletes (4). It is also essential to remove the distorting mirror of the leering male gaze, so frequently conveyed by the press in that period. Even today, this gaze discredits certain women's events, preventing them from being considered as “real sport.” The history of these early competitions, their chronological and geographical development, their organization and the backgrounds of the participants have yet to be studied, a task that requires considering them as a legitimate topic of study, despite the fact that they do not conform to the traditional definition of modern sports.

Of course, historians are extremely dependent on existing archives—mainly press articles—to write this story, and these offer scarce accurate descriptions of these sportswomen (8). There are few interviews and few leads to retrace their careers. Sometimes we do not even know their real names: one archived press photograph put online by the Bibliothèque Nationale de France in its digital library (Gallica) is simply titled “Portrait of a woman wrestler” (46). This considerably complicates the researcher's work. Yet this absence of information does not preclude a critical analysis of the media. Indeed, it seems important to question how this absence demonstrates the media's view of sportswomen, and to try to dig deeper to reveal the point of view of the practitioners themselves, outside of this distorting prism.

## Data Availability

The original contributions presented in the study are included in the article, further inquiries can be directed to the corresponding author.
